# Refugee Mental Health, Global Health Policy, and the Syrian Crisis

**DOI:** 10.3389/fpubh.2021.676000

**Published:** 2021-08-03

**Authors:** Kelso Cratsley, Mohamad Adam Brooks, Tim K. Mackey

**Affiliations:** ^1^Department of Philosophy & Religion, American University, Washington, DC, United States; ^2^School of Social Work, Columbia University, New York, NY, United States; ^3^Global Health Program, Department of Anthropology, University of California, San Diego, La Jolla, CA, United States

**Keywords:** refugees, refugee mental health, mental health, global health, global mental health, global health policy, health policy, Syria

## Abstract

The most recent global refugee figures are staggering, with over 82.4 million people forcibly displaced and 26.4 million registered refugees. The ongoing conflict in Syria is a major contributor. After a decade of violence and destabilization, over 13.4 million Syrians have been displaced, including 6.7 million internally displaced persons and 6.7 million refugees registered in other countries. Beyond the immediate political and economic challenges, an essential component of any response to this humanitarian crisis must be health-related, including policies and interventions specific to mental health. This policy and practice review addresses refugee mental health in the context of the Syrian crisis, providing an update and overview of the current situation while exploring new initiatives in mental health research and global health policy that can help strengthen and expand services. Relevant global health policy frameworks are first briefly introduced, followed by a short summary of recent research on refugee mental health. We then provide an update on the current status of research, service provision, and health policy in the leading destinations for Syrians who have been forcibly displaced. This starts within Syria and then turns to Turkey, Lebanon, Jordan, and Germany. Finally, several general recommendations are discussed, including the pressing need for more data at each phase of migration, the expansion of integrated mental health services, and the explicit inclusion and prioritization of refugee mental health in national and global health policy.

## Introduction

The most recent global refugee figures are staggering, with 82.4 million people forcibly displaced and 26.4 million registered refugees (1). The ongoing conflict in Syria is a major contributor. After a decade of violence and destabilization, over 13.4 million Syrians have been displaced, including 6.7 million internally displaced persons (IDPs) and 6.7 million refugees registered in other countries (1) (Table 1). Beyond the immediate political and economic challenges, the Syrian conflict has created a public health crisis and thus an essential component of any response to this ongoing humanitarian emergency must be health-related (2–4). This includes policies and interventions specific to mental health, given the unique and often acute mental health needs of IDPs, refugees, and asylum seekers. Compounding all of this is the COVID-19 pandemic, which has created additional mental health risks for this already vulnerable population (5), with the United Nations High Commissioner for Refugees (UNHCR) recently emphasizing that the pandemic has led to “widespread despair” among refugees (6)^1^.

**Table 1:** Global refugee crisis.

Forcibly Displaced	82.4 million
Registered Refugees	26.4 million
**Leading Countries of Origin**	
Syria	6.7 million
Venezuela	4.0 million
Afghanistan	2.6 million
South Sudan	2.2 million
Myanmar	1.1 million
**Leading Host Countries**	
Turkey	3.7 million
Colombia	1.7 million
Pakistan	1.4 million
Uganda	1.4 million
Germany	1.2 million

*UNHCR Global Trends: Forced Displacement in 2020*.

Although research on refugee mental health has only recently gained momentum, the emerging evidence describes high rates of mental disorder associated with trauma, stress, and cultural isolation due to forced migration, often met by a lack of adequate resources (7–10). While the available evidence on the mental health of Syrian refugees and IDPs remains incomplete and fragmentary, reports from multilateral agencies and NGOs point to high prevalence rates and a glaring scarcity of mental health services within countries impacted by the crisis (11, 12). This applies to the conflict zone within Syria, as well as the primary transit and destination countries in the Eastern Mediterranean region. There are also concerns about the burden of mental disorder and service accessibility in the European, Asian-Pacific, and North American countries where many Syrians have sought refuge or resettled (13).

This article provides an update and overview of refugee mental health in the context of the Syrian crisis, exploring opportunities for further research and strengthened health governance that can help drive the expansion of services. We first offer a brief background on relevant global health policy, followed by a short summary of recent research on refugee mental health. We then provide a selective analysis of the current status of research on refugee mental health, service provision, and health policy in several of the primary destinations for forcibly displaced Syrians. This starts with Syria itself and then turns to the leading destination countries of Turkey, Lebanon, Jordan, and Germany, examining the available evidence in order to draw out a set of provisional recommendations. Based on this review, we suggest that there is a pressing need for more data at each phase of migration, that fully integrated mental health services are a humanitarian necessity, and that refugee mental health must be explicitly included and prioritized in national and global health policy.

## Global Mental Health Policy

Mental health has recently taken on greater prominence within global health policy, in large part due to the policy instruments, technical guidance, and status reports of the World Health Organization (WHO). Many of these initiatives have direct relevance for refugees (Table 2). The WHO's groundbreaking *Mental Health Action Plan 2013–2020* (14), now extended to 2030 (19), adopts a rights-based approach to advancing universal health coverage, prioritizing improvements in health governance, the integration of services, promotion and prevention, and information systems. The *Mental Health Gap Action Programme* (mhGAP) provides planners, policymakers, and donors with a set of programs for scaling up services in low- and middle-income countries (LMICs) (20). The *mhGAP Humanitarian Intervention Guide* (mhGAP-HIG) was adapted and created to provide first-line management recommendations for non-specialist providers in humanitarian emergencies where access to specialists and standard treatment options are limited (21). And most recently, the WHO launched the *Special Initiative for Mental Health (2019–2023*) with the goal of expanding access to mental health services in twelve priority countries through new funding measures (22).

**Table 2:** Policy frameworks relevant to refugee mental health.

**WHO Mental Health Action Plan for 2013–2030** (14)
Objective 1: Strengthen effective leadership and governance for mental health
• Target 1.1: 80% of countries will have developed or updated their policies/plans for mental health • Target 1.2: 50% of countries will have developed or updated their laws for mental health
Objective 2: Provide comprehensive, integrated mental health, and social care services in community-based settings
• Target 2: Service coverage for severe mental disorders will have increased by 20%
Objective 3: Implement strategies for promotion and prevention in mental health
• Target 3.1: 80% of countries will have at least two national promotion and prevention programs • Target 3.2: Rate of suicide will be reduced by 10%
Objective 4: Strengthen information systems, evidence, and research for mental health
Target 4: 80% of countries will routinely collect and report mental health indicators every 2 years
**UN 2030 Agenda for Sustainable Development** (15)
Goal 3: Ensure healthy lives and promote well-being for all at all ages
• Target 3.4: By 2030, reduce by one third premature mortality from non-communicable diseases through prevention and treatment and promote mental health and well-being • Indicator 3.4.2: Suicide mortality rate
Goal 10: Reduce inequality within and among countries
• Target 10.7: Facilitate orderly, safe and responsible migration and mobility of people, including through implementation of planned and well-managed migration policies • Indicator 10.7.2: Countries that have implemented well-managed migration policies
**UN Global Compact for Safe, Orderly and Regular Migration** (16)
Objective 15: Provide access to basic services for migrants
**UN Global Compact on Refugees** (17)
Area 2.3: Expand health systems to facilitate access by refugees
**WHO Promoting the Health of Refugees and Migrants: Draft Global Action Plan, 2019–2023** (18)
Priority 1. Promote the health of refugees and migrants through a mix of public health interventions
Priority 2. Promote continuity and quality of essential health care
Priority 3. Advocate the mainstreaming of refugee and migrant health into global, regional and country agendas
Priority 4. Enhance capacity to tackle the social determinants of health and accelerate progress toward SDGs
Priority 5. Strengthen health monitoring and health information systems
Priority 6. Support measures to improve evidence-based health communication and counter misperceptions about migrant and refugee health

These initiatives have been highly influential and are particularly applicable to refugee mental health, as the majority of refugees invariably face challenges associated with scarce and inaccessible services. The WHO has built on this work by focusing on the specific needs of refugees in its development of technical guidance for refugee mental health provision in Europe (23). The WHO has also proposed a *Global Action Plan on Promoting the Health of Refugees and Migrants, 2019–2023* (18), which focuses on improving health services for this vulnerable population. It centers around several key priorities, including general health promotion, continuity of care, mainstreaming refugee health, the social determinants of health, monitoring and information systems, and health communication. The *Global Action Plan*, however, has not yet been formally adopted by the WHO or its member states.

The broader challenge is that there has been insufficient progress toward actually meeting the WHO's goals in global mental health. While on the national level most of the countries impacted by the refugee crisis have policy and legislation covering mental health services, there are enduring problems with insufficient funding and implementation, as evidenced by the WHO's own dedicated reporting mechanism (24) and its commissioned reports on refugee health and mental health (25–27). These deficiencies hinder the development, expansion, and allocation of mental health services for refugees, in LMICs and high-income countries alike. Despite the WHO's sustained efforts to support and provide services to refugees, including in Syria as well as a number of destination countries, a great deal more needs to be done.

The United Nations (UN) and UNHCR have of course been heavily involved in developing responses to the global refugee crisis and the Syrian conflict. This includes initiatives specific to mental health. The UN's *2030 Agenda for Sustainable Development* (15) foregrounds the issue of migration, particularly the human rights of migrants, and now includes mental health within its one health-related sustainable development goal as part of a broader target (SDG 3.4: “reducing premature mortality from non-communicable diseases through the prevention, treatment, and promotion of mental health and well-being”).

Consistent with SDG 10.7 (“facilitate orderly safe, regular and responsible migration…”), the UN recently adopted the *Global Compact for Safe, Orderly and Regularly Migration* (16) and the *Global Compact on Refugees* (17). Though not binding conventions, rather a closely related pair of cooperative frameworks, the former pact includes a commitment to providing “basic services” to migrants (objective 15), and the latter an expansion of health and mental health services accessible to refugees (area in need of support 2.3). These linked *Compacts* are grounded in the original 1951 *Convention Relating to the Status of Refugees* (28) and its 1967 *Protocol* (29), which require member states to guarantee refugees equal access to services. They are also consistent with the UNHCR's more recent call for countries to fully integrate refugees into their national health systems (30).

The UNHCR, for its part, has consistently supported a range of mental health services in many countries impacted by the ongoing refugee crisis (31), as well as commissioned reports on refugee mental health in key countries, including Syria (32). It has also developed its own technical guidance for mental health and psychosocial support (MHPSS) in humanitarian settings (33), working in conjunction with the WHO (34), and maintains a regularly updated MHPSS *Emergency Handbook* (35).

The longstanding concern, such initiatives notwithstanding, is that global health policy frequently runs the risk of leaving out important considerations that could otherwise help direct funding and support on-the-ground programming in global mental health. For a start, the SDGs only adopted a target with language specific to mental health after persistent advocacy on the part of civil society groups and academics (36); there are also lingering questions regarding the lack of coherence between the SDGs and other policy instruments in global mental health (37). The SDG framework has also come under criticism for insufficiently addressing the health of those impacted by conflict (38), and for failing to include refugee data in the regular reviews provided by member states (39). In addition, the UN's recent compacts on migration and refugees have been questioned for failing to sufficiently prioritize health (40). As the new Lancet Migration collaboration has emphasized (41), issues at the intersection of health and migration are far too often a secondary consideration and need to be explicitly included and prioritized in global health policymaking going forward. The same can be said for refugee mental health.

## Forced Migration and Mental Health

Reviews of research on the mental health of populations forcibly displaced by conflict have confirmed a substantial burden of mental disorder, although they have also consistently noted the lack of quality data and the marked heterogeneity of methodology and prevalence rates across studies. This variability has held even when systematic reviews have narrowed their focus and inclusion criteria. Early examples of such analyses include a review of studies comparing refugee and non-refugee populations, with refugees at moderately greater risk of mental disorder (42), and a review of research on refugees resettled in high income countries, suggesting much higher rates of post-traumatic stress disorder (PTSD) in refugees than in matched samples (43). Other reviews have found wide variations in prevalence rates, largely due to methodological factors (44), including those that have looked at the available data on longer term outcomes for refugees (45) and in settings of protracted conflict (46). Pooled estimates of prevalence have gradually become available. For example, a recent meta-analysis of international studies on refugees found high rates of PTSD (31.46%; 95% CI 24.43–38.5%), depression (31.5%; 95% CI 22.64–40.38%), and anxiety (11%; 95% CI 6.75–15.43%) (47), and a meta-analysis of research in Germany found similar rates of PTSD (29.9%; 95% CI 20.8–38.7%) and depressive symptoms (39.8%; 95% CI 29.8–50.1%) (48).

Amongst the many factors that complicate research in this area, perhaps most significant are the different phases of migration. Studies have typically focused either on the three phases of premigration, migration, and postmigration (49), or a five-phase approach that includes the following: pre-departure, travel, interception, destination, and return (50). But neither model is easily applied to the various risk factors associated with refugee mental health. Mental disorders are often the product of multiple factors across a variable time course, including preexisting genetic vulnerabilities as well as any number of social and environmental factors, consistent with the broader recognition of the social determinants of mental health (51). Forced migration affects many different types of people and is associated with a wide range of social stressors, ranging from the traumas of war to the difficulties associated with resettlement (52). Understanding exactly how these disparate risk factors combine to impact mental health during forced migration in specific populations is thus an ongoing methodological challenge.

That said, the different phases of migration have been increasingly linked to specific risk factors, especially as more is learned about the psychosocial stressors encountered during the postmigration—or destination—phase (53). A recent systematic review of the literature, paying particular attention to the complexity of resettlement in high income countries, has proposed an alternative model comprised of five phases: before travel, active travel, initial settlement in a host country, attempts at social integration, and any changes to immigration status (54). At each stage refugees appear to be susceptible to relatively specific types of risks; in addition, as this particular review suggests, they may also benefit from certain protective factors. Continued research on the different phases of migration, then, holds the promise of contributing to the development of more effective interventions and therapeutic techniques appropriate to this vulnerable population.

The premigration—or predeparture—phase typically involves a range of social and economic hardships, such as lost educational and occupational opportunities, as well as varying degrees of traumatic experience, including violence and torture. The data available on pre-migration tend to come from research on the long-term effects of trauma. For example, meta-analyses have found that the experience of torture and other forms of severe trauma, especially *cumulative* exposure, is predictive of higher rates of post-traumatic stress disorder (PTSD) and depression in refugees and displaced persons (44). According to recently updated WHO estimates, the burden of mental disorder in conflict settings is substantial, with high prevalence rates (22.1%; 95% UI 18.8–25.7%) across a range of conditions (55).

The migration phase encompasses the period of active travel, when individuals are between their place of origin and another location. This period of migration brings additional challenges, such as general fear and uncertainty as well as the harsh, often dangerous conditions of cross-border travel. Perhaps unsurprisingly, there is a dearth of literature on this phase, given the difficulties attendant to researching such a dynamic period within the broader process of migration. What work there is has largely relied upon surveys that include retrospective inquiries about stressful and traumatic experiences throughout the process of migration. For example, one study of refugees at various sites in Greece—entry points, camps, and informal sites—documented frequent reports of violent events experienced during the process of migration (over half of the sample was made up of Syrians) (56).

The interception phase refers to periods of temporary detention or interim residence, including time spent in refugee camps. Several studies have found that placement in detention centers is associated with poor mental health. For example, longer detentions have been associated with worse outcomes for detained refugees in Australia, leading to increased risk of PTSD and depression (57). Systematic reviews of the mental health of detained asylum seekers provide similar results, although the data is somewhat more equivocal (58, 59). The academic literature on mental health within refugee camps remains sparse, but a large survey of mental health service users in 90 camps across 15 LMICs indicates an increased risk of severe mental disorder—including psychotic disorders—in these settings (60). One study of Syrian refugees in a camp within the Attica region of Greece found high rates of depression, with female gender, having children, and longer stays in detention associated with increased risk (61). The gray literature is more extensive. Reports from NGOs, such as the International Medical Corps (IMC), have described high rates of depression and psychotic disorders in interim refugee camps in Turkey, Jordan, Lebanon, and Syria (11). Similar findings have been reported from an IMC survey of refugee camps in Greece (62), and a recent report from the International Rescue Committee (IRC) has called attention to the psychological distress caused by extended detention in refugee camps in Greece (63).

The postmigration—or destination—phase is the period during which refugees settle either temporarily or for the long-term in their intended location, most often in urban settings within host countries (it is also occasionally used to describe extended periods in refugee camps). This phase is the most accessible to research and can provide insight into prior stages of migration and the risks inherent to the process of applying for permanent refugee status and/or formal resettlement. Despite significant variation across studies, once again, systematic reviews have found that refugees resettled in both LMICs and high incomes countries have high rates of PTSD and depression in comparison to host populations (43, 64, 65). Many reviews have also identified postmigration social stressors that appear to moderate mental health outcomes. Such variables include financial hardship and socio-economic status, unstable housing, social isolation and loneliness, bigotry and discrimination, residency status, length of the asylum process, and cultural and linguistic barriers to integration (43–45, 66–69).

These findings underscore the complex challenges of resettlement. While reaching a destination country may provide some sense of stability, there are pronounced risks associated with this phase of migration (70). This includes the possibility of severe and chronic disorder, as the literature has slowly revealed. A number of studies describing the mental health difficulties faced by refugees from different countries of origin now living in Western countries—such as Australia and Sweden—reveal increased risks for psychotic disorders (71), prolonged grief (72), and suicide (73, 74). This is consistent with evidence of a general association between migration and psychosis, as there appears to be a dose-response relationship between number of social adversities—across the phases of pre-migration and post-migration—and psychosis (75). A detailed accounting of migration risk will require more research, though there has been significant progress. For instance, one increasingly influential model emphasizes the role of premigration trauma in the emergence of PTSD during the first 5 years of resettlement, followed by increased risk of depression resulting from difficulties in social integration and health care access after 5 years in the host country (76).

Regardless of phase of migration, the groups most vulnerable to mental health risks are women and children (77). Refugee women experience added vulnerabilities and stressors, including sexual, physical, and psychological abuse (78, 79), which contribute to greater risk for severe mental health conditions (80). Refugee women are also at a greater risk for intimate partner violence (81), which is also associated with risk of mental disorder (82). Reviews have confirmed the harmful effects that conflicts and forced migration can have on children and adolescents, as well as their caregivers (9, 83). In fact, a younger age at time of migration may put refugees at greater risk of mental health difficulties (7), and there appears to be a dose-response relationship between trauma exposure and severity of PTSD in children (84). In addition, data have described a disproportionate number of unaccompanied refugee children in psychiatric hospitals in destination countries (85). A growing evidence base more generally points to the need for increased efforts to address the mental health of refugee children going through the process of resettlement (86–90).

In terms of therapeutic interventions, for refugees of all ages and backgrounds, much is still not known. Consistent with the general lack of data, research on interventions for refugee mental health has been slow to develop (91). Reviews of the available evidence have found that cognitive behavioral therapies are the most empirically supported psychosocial treatments for PTSD in refugees (92–94). There are also promising new research programs underway. Of particular note is an initiative that is deploying components of the WHO's mhGAP package to support the expansion of mental health services for Syrian refugees in European and the Middle Eastern countries. Under the auspices of the STRENGTHS program, based out of VU University Amsterdam, a relatively adaptable psychological intervention delivered by non-specialists—as a form of task-shifting—is currently being tested in different formats in a range of settings (95). This includes research on an internet-based version of the intervention in Germany, Sweden, and Egypt (96), and peer-based delivery in the Netherlands (97).

## Syria

Much of what is known about mental health in conflict settings and forced migration applies to the ongoing crisis within Syria. Approximately 6.7 million Syrians have been internally displaced, with an additional 6.7 million displaced across borders (1) (Table 3). The conflict has created a public health emergency for those who continue to reside in the country, including the internally displaced (98), as well as those who have fled as refugees. This has had devastating effects on the mental health of Syrians, creating an increased disease burden while compromising the country's capacity to provide health and mental health services. Targeted attacks on hospitals and medical professionals have severely impacted health provision (99–101); early in the conflict this included direct bombardment of psychiatric hospitals in Aleppo (102). Such attacks, widely reported in the popular press and the gray literature (103–105), have made their way into the research literature (106, 107), including powerful first-hand accounts of the impact on both providers and patients (108). The COVID-19 pandemic has further taxed remaining health services and exacerbated vulnerabilities among those forcibly displaced (109), contributing to the growing burden of mental disorder (110).

**Table 3:** Forcibly displaced Syrians.

Internally Displaced	6.7 million
Displaced Across Borders	6.7 million
**Leading Host Countries**	
Turkey	3.6 million
Lebanon	0.87 million
Jordan	0.66 million
Germany	0.61 million

*Registered refugees only*.

*UNHCR Global Trends: Forced Displacement in 2020*.

*UNHCR Refugee Data Finder*.

There is a limited amount of quality data available, but existing evidence points to widespread worsening of preexisting mental disorders and the emergence of new disorders due to exposure to violence and displacement (32, 111–114). Relatively early in the crisis it was estimated that more than 2 million people were suffering from depression and anxiety and over 350,000 from severe forms of mental disorder (115). Other research has estimated 2.2 million cases of PTSD and 1.1 million cases of depression (with a depression rate of 13.3%) (116); another study found high rates of PTSD (31.8%) in internally displaced Syrians (117). NGOs working in Syria have also described high rates of depression and anxiety (11), and case studies of displaced Syrians with mental health conditions have started to appear, from both within Syrian and other countries (118, 119). The unresolved nature of the conflict has created prolonged exposure to stressful and traumatic experiences (120), which suggests that psychiatric morbidity can only have steadily increased over time. Indeed, a recent large online survey found high rates of PTSD (36.9%) (121), and another recent survey from the NGO Syria Relief found PTSD in the near entirety of a sample of IDPs in the Idlib governorate (12).

Children and families, especially women and girls, have been disproportionately impacted (113, 122). A survey of caregiver reports from refugee camps in northern Syria has suggested that nearly half of displaced children exhibit the symptoms of emotional and behavioral disorders (123), and a school-based study in Damascus and Latakia reported high rates of PTSD (35%), depression (32%), and anxiety (29%) (124). One recent large study found very high rates of PTSD (53%) in schoolchildren in Damascus (125), and a small case file review of children in schools around Idlib described a range of mental health difficulties (126). Women have been found to be at increased risk of a number of vulnerabilities, including depressive symptoms (127) as well as sexual and gender-based violence and barriers to health care (128, 129).

There continues to be limited research from within Syria, especially regarding access to MHPSS, indicative of ongoing, extreme insecurity in the country. A major contributor to the public health emergency in Syria is service scarcity due to damaged healthcare infrastructure, including mental health services. Prior to the conflict, the country benefited from a relatively effective health care system (101). It is also notable that Syria has a relatively new national mental health strategy, from 2014, not to mention dedicated mental health legislation (11, 24). But given the ongoing conflict, these policies remain only partially implemented. Efforts have been made to rebuild and strengthen the devastated healthcare system, but the ongoing challenges are difficult to overstate. Only 48% of public hospitals and 48% of private hospitals are fully functional, with half of all health professionals still residing outside the country (130).

The public psychiatric hospitals that were initially closed or had their capacity significantly limited due to the conflict are now functional (130). However, the mental health workforce remains diminished. Figures from the WHO on Syria's mental health capacity include 1,931 mental health professionals, with 0.37 psychiatrist, 1.07 nurses, 1.07 psychologists, and 0.08 social workers per 100,000 population (24). This staffing shortage represents a weakened capacity for case management and treatment by specialized mental health care providers while the burden of mental disorder increases. Furthermore, there is a lack of community based mental health services, making it difficult for services to reach those living outside of large cities.

In response to acute mental health coverage shortages, there have been efforts by multilateral agencies and local and international NGOs to help rebuild and expand service capacity. The WHO has supported the delivery of mental health services in more than 150 primary and secondary health centers in 11 different governorates, staffed by over 1,000 WHO-trained non-specialist general practitioners, 2,000 health workers, and more than 60 psychologists (131). New psychiatric inpatient units have been set up by the WHO in general hospitals in the cities of Damascus, Hama, and Latakia, and more than 100,000 people were estimated to have received mental health consultations in 2016 (excluding those not tracked by the WHO system). The WHO has also supported community-based initiatives, including mental health services in community centers in Aleppo, the distribution of mental health manuals in inaccessible areas, and the development of a school-based instructional package. By 2018, the WHO was reporting that over 400 primary health centers and community centers were providing MHPSS services (132). The WHO has also been working on a project to deploy mobile psychological clinics in northern Syria to address the needs of IDPs who live in remote areas where traditional inpatient and outpatient services are not available (133). Similarly, UNHCR has supported 130 Community and Satellite Centers throughout the country which provide MHPSS services, in large part through a trained volunteer workforce (134).

Many international and local NGOs have contributed to this important work [for reports on some of these initiatives, see (135–137)]. This includes Syrian NGOs, largely operating from outside the country, such as the Syrian Association for Mental Health, the Union of Syrian Medical Care and Relief Organizations, and the Syrian American Medical Society, as well as charities such as Al-Sham and Balsam (138). Despite these efforts, the need for mental health services in Syria continues to massively outstrip available resources. A large number of Syrians with mental health conditions receive no treatment at all, and the pandemic is further aggravating the situation. With the seeming intractability of the Syrian conflict, novel service-level and policy-based interventions need to be considered in order to improve public health conditions within the country, including services for the internally displaced, as well as to improve health and mental health coverage for refugees who transit to other countries.

## Destination Countries

Given the number of people forcibly displaced from Syria, and the association between refugee status and mental health difficulties, it is necessary to evaluate the resources available to Syrians who leave the country. Many of the challenges described in the literature on refugee mental health afflict Syrian refugees traveling to and residing in other countries. Syrian refugees living both in camps and amongst the general population in transit and destination countries suffer from high rates of mental distress and disorder. Mental health difficulties have been identified as the third greatest health need for Syrians displaced to neighboring countries in the region, behind communicable diseases and women's health (139). A review of Syrians in neighboring countries found high prevalence rates of mental disorder, and also included data on individual risk factors, singling-out traumatic experiences and a prior history of mental disorder, as well as reviewing research on access to MHPSS, identifying financial costs and socio-cultural factors as key barriers (140). And a recent systematic review of research on Syrians in countries spread out across both the Middle East and Europe found weighted prevalence rates of PTSD (23.4–83.4%), depression (20–44.1%), and anxiety (19.3–31.8%) (141).

As previously noted, methodological differences account for a great deal of the heterogeneity across studies, but research on refugee mental health more generally also reveals significant variation between destination countries (45), as culture and context appear to play a significant role (112, 114). There are some noticeable demographic trends, which underscores the importance of ecological validity in research on refugees. For instance, those arriving in Europe tend to be young men [e.g., (48)]. But variation is also seen more generally in service availability, access, and usage. Research on mental health consultations in primary care centers within refugee camps across several countries and regions has revealed a wide variation in rates of visits and types of disorder (60). The specific destination of forcibly displaced people may significantly impact prevalence, morbidity, and service utilization, in addition to the role of the particular social, cultural, and economic backgrounds of refugees.

This section, then, provides a brief overview of the status of refugee mental health research, service availability, and relevant health policy in several key destination countries, with a specific focus on Syrian refugees. This starts with the leading destinations in the region, Turkey, Lebanon, and Jordan, and then extends to the country that has received the largest influx of refugees traveling across Europe, Germany. This selective approach is guided by the available evidence in order to explore broader responses in service provision and health policy and governance. There are other countries in the Middle East and Europe that should be the focus of similar analyses. According to UNHCR estimates, Iraq (247,305), Egypt (132,748), and other North African countries (31,657) have been major destinations for Syrian refugees and asylum seekers (142). In Europe, other notable destination countries include Sweden (114,609), Austria (54,903), and the Netherlands (22,447) (143). In North America, Syrians have been resettled over the last 5 years in Canada (44,620) and the United States (21,725) (144, 145).

### Turkey

Even with its own ongoing political upheaval, Turkey continues to be the main destination for Syrian refugees, with over 3.6 million registered (146). Approximately 98% of refugees reside in urban and rural areas across the country, while <2% live in the seven remaining “temporary accommodation centers” (147). Consistent with broader estimates, research specific to Syrian refugees in Turkey describes high prevalence rates of mental disorders. For example, a study among Syrians living in a tent city found that more than a third had PTSD (148); another estimated extremely high rates of PTSD (83.4%) and depression (37.4%) in a refugee camp (149). A WHO household survey across 15 provinces revealed reports of severe or extreme depressive feelings in 17% of adult respondents (150). In Istanbul, a recent survey found high rates of PTSD (19.6%), depression (34.7%), and anxiety (36.1%) (151). This study is consistent with other research in that it identifies several key predictive variables, some of which concern aspects of post-displacement. These include: female gender, prior trauma, financial challenges, and ongoing needs for social support, as well as a perceived lack of safety, legal protection, and justice. There is also evidence of high rates (23.7%) of mental disorder in children and adolescents in Istanbul (152).

There have been reports that mental health services are difficult for refugees to access in Turkey (11, 153). In a separate analysis of the same sample of Syrian refugees in Istanbul just mentioned, it was revealed that the vast majority of individuals who screened positive for mental disorders did not seek treatment (154). Respondents to the survey mentioned a range of barriers, including treatment costs, stigma, lack of knowledge of the health system, and the belief that symptoms would resolve over time. These findings highlight the need for community based MHPSS programs that can address these barriers and reduce the treatment gap.

Nothing on the legislative or policy level bars healthcare access. Registered refugees have the legal right to make use of the country's universal health care system under the “Temporary Protection Regulation,” although there are practical barriers such as delays in enrollment, during which one is ineligible for anything other than emergency services, as well as obstacles related to travel, language, and basic understanding of the national health scheme [all of which impact Syrian refugees (155–158)]. There is an official national mental health policy, though no dedicated legislation is in place (24). Indicative of the broader challenge of implementation, the national policy calls for increased commitment to community-based and integrated services. But neither of these goals has yet to be met in Turkey, as in most countries (24).

The WHO has partnered with the Ministry of Health and multiple international donors to provide health services to displaced Syrians. As part of Turkey's Refugee Health Programme, over 180 Migrant Health Centers have been supported throughout the country, which are also the site for training Syrian health workers with the goal of integrating them into the national health system (150, 153, 159). Surveys suggest that Syrian refugees primarily rely upon hospitals for their health needs, but there are plans to continue to expand the number of refugee health centers so as to increase access and utilization (150). The WHO also operates cross-border health services in northwest Syria out of a field office in Gaziantep (153). This includes maintaining a medical supply chain, deploying mobile clinics, and offering primary health care services in WHO-supported facilities. Few of these facilities offer mental health services, and there is a staffing shortage of psychiatrists and other mental health professionals. In response, the WHO has trained non-specialist health workers and community outreach workers with the mhGAP program.

### Lebanon

The situation in Lebanon is not dissimilar, although the country faces a very particular set of challenges due to recent political unrest, an economic crisis, and longstanding support of a large population of Palestinian refugees. The complexity of the health system also brings with it special challenges. There are now ~1.5 million Syrian refugees—both registered and unregistered—residing in Lebanon, not to mention 35,000 Lebanese nationals and 34,000 Palestinian refugees who have all fled Syria since the start of the conflict (132, 160). The majority of Syrian refugees reside throughout the country in informal settlements, often under poor conditions in tent cities situated within already impoverished communities. Lebanon has never signed on to the 1951 *Refugee Convention* and so no formal camps have been established, although limited social and health-related protections are granted to refugees based upon an agreement with UNHCR (161). Such arrangements have left refugees in a precarious position within the country, creating challenges to the health system (162, 163), including mental health programming (164, 165). The recent explosion in Beirut, which damaged several hospitals and stores of medical supplies, only worsened this state of affairs (166).

There are numerous reports of a substantial burden of mental disorder, particularly PTSD, in the Syrian refugee population in Lebanon, both from the gray literature and academic research. For example, studies have found high rates of PTSD in Syrian refugees (167, 168). Since the start of the Syrian conflict, there appear to have been significant increases in rates of depression (169) and psychiatric hospitalization (170). Research has also started to focus on the impact of trauma and living conditions on refugee families: Syrian women exposed to war and conflict-related events tend to suffer from psychological distress which can, in turn, lead to negative parenting and psychosocial difficulties in their children (171). These findings highlight the intergenerational impact of war, conflict, and displacement. There is also emerging evidence confirming the deleterious effects on health and mental health of the substandard living conditions endured by Syrian refugees in the informal tent cities scattered across the country (172).

The health system in Lebanon has been overburdened by the sheer scale of the influx of refugees, although a number of initiatives between the Ministry of Public Health (MoPH) and its international partners have tried to bolster service provision. Due to the highly privatized nature of the preexisting health system, the needs of refugees have been primarily served through hospitals and clinics funded and operated by the WHO, UNHCR, and various NGOs (173). Following the start of the Syrian crisis, the Lebanese government worked with UNHCR to initiate a humanitarian response plan (174), and the MoPH developed a strategy for the health sector (175). These plans prioritized improved access of services for refugees, including public services provided by the Ministry of Social Affairs, predominantly centered around the expansion and support of public health centers (PHCs). Since then, the WHO has reported a number of positive developments in the work of PHCs, including the distribution of free vaccinations and the subsidized provision of medication and other essential health services (132). But despite these efforts, accessing care continues to be a problem for many Syrian refugees given the remaining financial barriers (163), especially for the treatment of non-communicable diseases (176, 177).

Many of the same challenges can be found within the mental health system in Lebanon, which until recently was predominately privatized and remains underfunded, understaffed, and largely restricted to urban centers (24, 164, 165). But here too there has been a coordinated national response to the Syrian refugee crisis, supported by multilateral agencies and NGOs. Spurred by an early report from the UNHCR (178), the MoPH convened a task force with UNHCR and the WHO to help guide MHPSS expansion, as well as initiating a National Mental Health Programme in conjunction with UNHCR, WHO, UNICEF, and IMC (179, 180). Ultimately a new national mental health plan was adopted in 2015 (181), with a commitment to improve services for displaced populations, including Syrian refugees. While not fully implemented (24), the WHO has reported progress on efforts to expand mental health services in PHCs, including the provision of basic MHPSS, training of staff on mhGAP materials, and the creation of a mental health data registry shared by select PHCs, private clinics, and hospitals (132).

There are also reports of innovative initiatives in health and mental health service delivery. Informal Syrian healthcare workers appear to be increasingly active in the provisioning of basic health services (182, 183). Lebanon's first early intervention in psychosis service was established out of the American University of Beirut (184). Innovations also include the use of e-mental health (telepsychiatry or telemental health) interventions, including WHO's “step-by-step” intervention package, which has been trialed with Syrian refugees—a population with marked vulnerabilities but high accessibility to smartphones and the internet (185)—with promising results (186). The COVID-19 pandemic has provided additional reasons to expand such services in Lebanon (187). For its part, the MoPH, which has often been encouraged to take a stronger leadership role in health governance (162), continues to push ahead with national-level plans, including MHPSS-specific responses to both the COVID-19 pandemic (188) and the explosion last summer in Beirut (189). These recent events may in fact represent an opportunity to build upon the existing collaborative governance model in order to further advance reforms to the mental health system in Lebanon (190).

### Jordan

In many ways Jordan's health system is fairly robust and better situated to address refugee mental health than many other countries, but here too there are ongoing challenges. It has been the destination for 1.36 million Syrian refugees—registered and unregistered—with 90% living outside camps in urban areas and ~140,000 in the two camps of Za'atari and Azraq (191). Similar to other destination countries, high rates of mental distress and disorder have been reported in the Syrian refugee population, with particular impact on children and adolescents (11, 192, 193). For example, a small study of Syrian children described feelings of loneliness and depression in a quarter of a sample of respondents (194), while a school-based study in the cities of Mafraq, Sahab, Ramtha, and Zarqa found moderate to severe PTSD in a substantial portion of the sample (31%), with higher rates in girls (195). There is also relevant research on adult Syrian refugees: a review of the available data found high rates of mental health difficulties across several studies (32.9%) (196). More recent studies recorded high levels of perceived stress and the presence of depressive and anxiety symptoms in Amman (197), as well as prolonged grief—associated with severe mental disorder—in the Azraq refugee camp, associated with severe mental disorder (198).

The government of Jordan developed a comprehensive strategy in response to the Syrian crisis that included a focus on strengthening the health sector (191, 199). An emphasis was placed on ensuring equitable access to health services so that registered Syrian refugees, outside of the camps, could access the full range of primary and secondary services offered by the public health system. Within mental health, the response plan was consistent with the preexisting national mental health policy (200) in its prioritization of the integration of mental health in primary care and the expansion of MHPSS. There have been challenges to the full implementation of the country's health and mental health policy, however.

In particular, various obstacles have dramatically impacted access and service usage. According to some estimates, more than half of Syrian refugees that need medication or other health services cannot access such services (201). A widely reported increase in co-payment fees created a financial barrier to treatment (193, 202, 203), and surveys found that the financial costs of health services are a problem for Syrian refugees (204). The government increased the subsidy on health services for refugees in 2019, but there have been indications that Syrian refugees were largely unaware of this change (205). These challenges related to primary healthcare access inevitably impact mental health service provision. Different forms of mental health interventions are increasingly available, both in inpatient and outpatient settings, but there have been significant gaps in coverage for refugees. The government of Jordan has started various social services initiatives to address these types of challenges, such as cash transfer programs for families, with some reported success (132).

### Germany

Germany has been the destination for many forced migrants, including 605,338 Syrian refugees now registered in the country (143). When large numbers of refugees started crossing into Europe in 2015, several German cities were key landing sites; for example, a great deal of strain was placed on health and social services in Munich (206). Early research initiatives soon began, including work on the mental health of refugees in Dresden (207). Subsequent research has had specific relevance to Syrian refugees. For example, two small studies in the state of North Rhine-Westphalia that focused on refugee children, both Syrian and Iraqi, found elevated rates of depression, anxiety, attention deficits, and withdrawal in relation to comparison samples (208). There is an ongoing study of adult Syrian refugees in Erlangen that found diagnosable conditions in a third of the sample (209); a recent follow-up with half of the original participants confirmed that these rates have remained substantial over time (210). High prevalence rates have also been reported in Halle (211). As previously mentioned, a meta-analysis has confirmed high prevalence rates in studies within Germany of refugees and asylum seekers from many different countries of origin (48). And a recent review of postmigration contextual factors has identified a range of risk factors, such unresolved asylum status, separation from family, and discrimination (212).

Addressing the burden of mental disorder has been difficult given legal restrictions on the provision of health services to refugees. By the terms of Germany's Asylum Seekers Benefits Act (213), new arrivals can only access “essential” health services for their first 15 months. Such services are typically restricted to acute conditions, effectively excluding any number of physical and mental illnesses. The law thus represents a significant barrier to accessing health and mental health services, which has led to sustained criticism (214). The UN has issued a report critical of this policy and coverage position (215), as did a group of academics and civil society organizations (216), characterizing it as a failure to protect the right to health that requires immediate reform. The German government issued a formal reply to the UN (217), maintaining that the law does ensure access to basic health care. This past year the government also issued a new global health strategy (218), although this too has come under criticism for failing to include any consideration of issues relating to migration (219).

Beyond the initial period of resettlement, refugees ultimately can gain the right to enroll in Germany's universal health care system, but even then it is not always easily accessed or utilized. Struggles with language, unemployment, and acculturation can represent significant obstacles for refugees to access and benefit from mental health interventions (220). Comprehensive data has been slow to become available, but there has been a clear need for enhanced service development specific to refugee mental health, particularly the provision of psychosocial therapies for trauma-related disorders (7). Recently, consistent with the growth of research on refugee mental health more generally, Germany has been the source of promising research on new service models and therapeutic interventions that could be scaled-up as a response to the longstanding access problems.

The Charité University Hospital in Berlin has been at the center of efforts to design, research, and implement mental health services for refugees. They have partnered with NGOs to provide services in Jordan, and in Berlin offer treatments from a specialist outpatient clinic for refugees (221). There are also a number of other research-based initiatives underway, including the development of a new screening tool (222), interventions for children (223), and multidisciplinary treatment packages for adults (224). The RefuKey initiative attempts to lower barriers to treatment through the provision of culturally appropriate interventions; research on the model has included Syrian refugees (225). There is also large, multicenter study of collaborative care model, tailored for Syrian refugees, that has just started being investigated (226). All of these treatment models, if proven effective, could serve as the basis for scaled-up interventions in similar high-income settings.

## Discussion and Recommendations

In order to address the problems associated with inadequate mental health services for Syrian refugees, as well as the internally displaced within Syria, a range of responses need to be considered. Here we focus the discussion on a set of provisional recommendations in several key areas; namely, future research priorities, capacity building and health systems strengthening, and national and global health policy (Table 4).

**Table 4:** General Recommendations.

Research Priorities
**Enhance data collection**
Track mental health correlates and outcomes throughout the migration process, collecting disaggregated data on gender, age, and family status
**Develop culturally specific mental health instruments**
Adapt assessment measures to refugee populations, including culturally specific idioms of distress and mental disorder
**Expand research on therapeutic interventions**
Research mental health interventions designed for forcibly displaced populations, with a focus on women and children
**Capacity Building and Health Systems Strengthening**
**Increase funding from regional and international donors**
Target underfunded mental health services for refugee populations to promote equitable access across destination countries
**Improve social and economic conditions**
Provide interventions that address socio-economic factors among the forcibly displaced, along with increased resources for local communities
**Integrate psychiatric care with broader health and human services**
Increase availability of psychiatric care and remove barriers to diagnosis and treatment, while providing clinically effective and culturally sensitive services
**National and Global Health Policy**
**Ensure equal access to health services for migrants**
Make health policies and health system implementation compliant with the UN's 1951 *Refugee Convention* and consistent with its recent *Compacts*
**Increase international pressure on the Syrian regime**
Monitor, reduce, and prevent targeted attacks on healthcare facilities and medical personnel
**Strengthen global governance**
Leverage policy instruments to influence international donors, national health ministries, and program implementers

### Research Priorities

There is a pressing need for more data. Research has only just started to examine the nature of mental health problems in refugee populations, and a number of questions remain unanswered that, with additional clarification, could help guide the expansion and improvement of relevant services [also, see (2, 227)]. For a start, given the variability in existing findings, future research should aim for more rigor and consistency, including a longitudinal focus on the impact of protracted conflict on both refugees and the internally displaced (45, 46, 64). Part of this should include disaggregated data that better reflect the role of gender, age, and family status (228), as well as a new focus not just on mental health risks but also on potential protective factors (54). For example, there is interesting new research on post-traumatic growth in refugees, including data suggesting that higher levels of education are associated with more positive psychological and social changes following trauma in adult Syrian refugees in Istanbul (229).

It is important to note that instruments used to measure mental health are largely based on Western notions and do not include culture-specific idioms of distress and conceptions of mental disorder (45, 64). A process of cultural adaptation and testing of mental health screening tools is needed when working with refugees to ensure reliability and validity of mental health symptoms being measured (230). In addition, there is need for increased attention to variations in individual responses to trauma. Consistent with research on the significance of events that follow on, after the fact, from violence, armed conflict, or natural disaster, the classical conception of PTSD does not appear to sufficiently account for the role of social and contextual factors in humanitarian crises (119, 231, 232).

The complex relationship between preexisting vulnerabilities and exposure to stress and trauma, across the various phases of migration, remains one of the most challenging research questions. Clearer and more conclusive evidence would not just help with clinical diagnosis and treatment, but it could ultimately assist with the structuring of service provision. It would inform our understanding of the patterns of variation that have started to emerge from the existing data, thus opening up the possibility of more targeted interventions. For instance, there are significant between-country differences in the prevalence rates of various disorders, service use by gender, and the percentage of children in treatment (11, 45). Again, this will require a better understanding of the role of social and cultural variables at each stage of displacement, with many of these variables also mediated by the policy environment (e.g., type of national health system; healthcare access specific to refugees; financial costs; etc.). Generation of more robust data that takes into account the complete pathway of forced displacement can help in the development of interventions—and health systems—that are responsive to the causes of distinct profiles in prevalence and service utilization.

What would inform these kinds of consideration is improved tracking and measurement of mental health correlates and outcomes throughout the migration process. There is no shortage of organizations involved that could strengthen their data collection systems to better feature mental health variables. For example, partnerships between the UNHCR and other agencies such as the IOM and WHO could recommend that member states commit to sharing much needed epidemiological and health systems data relating to Syrian refugees in a common, transparent data schema. Greater access to cross-border data could enable better health outcome and system research, which could, in turn, inform the design of future interventions and the identification of key challenges that transit and destination countries could collectively prioritize.

All that said, the barriers to research in humanitarian settings are substantial, and the topic of mental health—which faces the challenges of underdiagnosis and stigma—in relation to the Syrian crisis is no different. It is difficult to carry out studies in this context; hence the interest in research from a distance—e.g., epidemiological modeling (116). Research may also hinder immediate relief efforts, while offering no guarantee that it will lead to effective interventions, particularly when adequate funding to carry out these interventions is not available. Increased engagement with local populations through principles of community-based participatory research can help address some of these concerns, potentially leading to more sustainable and effective interventions (233). This implies that health data partnerships should extend well-beyond multilateral agencies, including NGOs, community groups, and other health and humanitarian stakeholders.

The research on therapeutic interventions for forcibly displaced populations remains in its early stages, although progress is starting to be made. As mentioned, several continuing research programs should soon provide more substantial evidence of the effectiveness of various psychosocial treatments. Some of this work specifically involves Syrian refugees. Of particular note is the STRENGTHS program currently being trialed in a number of different settings (95). But there is clearly room for much more research in this area. Others have sounded calls for research in high income countries to focus on several particular issues, including task-shifting approaches that rely upon peer-led interventions, telepsychiatry and e-mental health interventions, and more basic science investigating the neurobiological mechanisms involved in the treatment of PTSD (91). There is also a need for more work on developing effective interventions for displaced women and children (9, 93).

### Capacity Building and Health Systems Strengthening

Much of the work required to rebuild and expand the health system within Syria will ultimately be political in nature, taking up issues that fall outside the scope of the present discussion. Most immediately, putting an end to the targeted attacks on healthcare facilities and medical personnel demands sustained international pressure. But there are other aspects of this work at the service level that are directly relevant to issues discussed here. Improving the provision of mental health services in refugee camps, especially in the northwest of the country, is highly dependent upon the continuing operations of multilateral organizations and NGOs in Syrian and neighboring countries. The WHO's cross-border work from Turkey is a good example of this. New, innovative responses are also necessary, for instance, modeled on initiatives such as the Brotherhood Medical Center, a privately funded clinic in the border town of Atimah which provides maternal and child services (234, 235).

In the leading destination countries, there are any number of ways of expanding and enhancing mental health services that should be under consideration. This starts with increased funding from international donors. Health services for refugee populations remain heavily reliant on the continued support of public and private funders, particularly in countries such as Lebanon. The influence of multilateral agencies such as the WHO is vital, but there has also been an increased awareness of the need to better coordinate the funding and provision of humanitarian programs across all of the organizations involved (236). These efforts need to be led by national health ministries in order to build strong and sustainable health systems, especially in the wake of humanitarian crises. This can help ensure that national health systems respond effectively to incoming refugees, given the domestic policy backdrop and the particularities of the country and culture. But key transit and destination countries should not do this alone; they should be supported by multilateral partners—as well as high-income countries that currently do not admit adequate numbers of refugees—in order to establish coordinated global governance that is responsive to refugee health and humanitarian needs more generally. The bottom line is that the financing of health services for refugees should come at both the national and supranational levels; there are a number of innovative funding mechanisms that need to be considered (237).

As repeatedly emphasized, social variables are key to refugee mental health and therefore appropriate interventions should be central to all service provision. Many of the contributors to poor mental health increasingly appear to be social and economic, such as poverty, unemployment, and discrimination, all exacerbated by war, conflict, and displacement. These variables are modifiable and thus appropriate socio-economic interventions should be incorporated into, or at least aligned with, mental health (49, 238). Cash transfer programs are an example of this type of approach (at use in destination countries such as Turkey, Lebanon, and Jordan). Efforts to improve basic living conditions are likely to improve health and mental health outcomes; this is particularly important when refugees all too often subsist under horrible conditions often without even the most basic amenities (172). There is another side to this as well, which is the tense dynamic that often develops between local populations and refugees. Ensuring that the local population has sufficient access to social services, economic support, and health services can help ameliorate what is otherwise a difficult situation given limited resources (239).

The importance of postmigration social factors also strongly suggests that health and mental health programming should prioritize service configurations that are fully integrated. In order for mental health services to be effective they must be linked to other health services, as well as community resources when possible, such as social services. Such a configuration has the potential to make interventions more accessible, given the broader reach of integrated services, as well as the possibility to circumvent the stigma frequently attached specifically to mental health services. But the fact remains that most countries have not successfully implemented integrated mental health provision, even when it is dictated by national and global health policy (24). The emphasis on integrated care also suggests that basic psychosocial interventions, as well as substance abuse treatment (240, 241), should be part of the clinical treatment package, and that community-based care is essential. This will require additional training of staff, especially in child psychiatry (242), as well as the continued development of non-specialist, task-shifting initiatives. Emphasis should be placed on tailoring clinical interventions to the cultural and contextual particulars of specific refugee populations, such as resettled Syrian refugees (243). To support this, more should be done to increase community participation in both the design and implementation of health services for the forcibly displaced (244).

The need to better integrate psychiatric care with broader health and human services is a continuing challenge for all health systems, including those in high income countries where Syrian refugees have been resettled. The challenges of fragmented services, and overall scarcity, also apply, although to a somewhat lesser extent. Where basic mental health systems are in place, the challenges tend to center on the removal of barriers to diagnosis and treatment and the provision of more clinically effective and culturally sensitive services. For instance, even where services are readily available there can be significant delays in the process of enrolling in health systems, a problem that can be problematic for refugees requiring immediate mental health care following the stressful and traumatic experiences associated with migration. The literature makes it clear that the mental health needs of refugees in high income countries require social support in various forms, such as promoting social integration, reducing barriers to treatment, especially financial, and increasing engagement with services through improved cultural competence on the part of practitioners (245).

### National and Global Health Policy

On the policy front, there are clear opportunities for improved governance. The guiding principle throughout should be a commitment to ensuring equal access to health services for migrants of all types. In practice, this means making national health policies, and health system implementation, compliant with the UN's 1951 *Refugee Convention* and consistent with its recent *Compacts*. This will take sustained advocacy and negotiation on the national, regional, and global level. A key challenge will be effective policy implementation and the monitoring of commitments, as health services remain out of reach for many refugees even in countries with universal health coverage in their constitution or national legislation. And of course, just as refugee health remains a challenging area in which to press for accelerated and substantial progress, a narrower focus on refugee *mental* health has in many ways proved to be even more of a challenge.

Within Syria, little progress can be made without international pressure. The debates in global health policy have centered on several key issues. The first concerns the impact of sanctions on the provision of humanitarian aid, particularly in the context of responses to the COVID-19 pandemic (246–248). Closely related, there has been concern about the restrictions placed on the work of NGOs—and the bilking of humanitarian aid—by the Syrian government (249, 250); this has led to criticisms of the potential complicity of the UN's Office for the Coordination of Humanitarian Affairs (251). Lastly, the targeted attacks on healthcare facilities and medical personnel continue to devastate health system infrastructure, despite attempts by both the UN and WHO to institute monitoring systems and establish UN “safe zones” to track and help prevent such attacks (101). Bold new responses are needed to address, and ultimately resolve, these issues, including approaches that have received less attention to date. For example, regional legal mechanisms specific to IDPs, such as the Great Lakes Pact and Kampala Convention, may serve as models for developing governance frameworks that can be applied to forced migration in Syria and neighboring countries (98).

Specific to multilateral institutions, the response of the UN and WHO needs to focus on the situation within Syrian and across destination countries, in the region and elsewhere. As noted at the outset, global health policy frameworks must begin to prioritize refugee health and mental health—explicitly and immediately—as a transnational health and humanitarian issue (41).There are some indications that mental health interventions are increasingly recognized as important components of humanitarian response (252), and the UN's *Compacts* and the WHO's *Global Action Plan* can help set constructive terms for further progress on this necessary, long-overdue shift. Future iterations of the SDGs, the most influential framework currently guiding investment in international development, should incorporate additional targets or shared indicators that measure progress toward refugee-specific mental health objectives (e.g., the provision of mental health services to refugee populations, or lowering the rate of PTSD). This is consistent with early calls to incorporate a mental health target that addresses service provision (253), as well as the more recent proposal from the Lancet Commission on Global Mental Health and Sustainable Development for a range of new mental health indicators (254).

Progress on the SDGs has been slow, and there are concerns that refugees have not only failed to benefit but have been left out entirely of many of the voluntary status reports submitted by member states (39). But it remains a promising, and influential, global agenda, with the potential for making necessary adjustments in order to address neglected targets—such as refugee mental health—if more purposefully constructed. There have been proposals, for instance, to leverage the SDGs for increased attention to the needs of children (255), and refugee women (256). The WHO's *Global Action Plan* also holds promise for refugee mental health, but at this stage it still requires formal adoption, followed by sustained commitment to implementation (257). Incorporating the *Global Action Plan* into an SDG indicator for progress on refugee health might be another way to change this soft law mechanism into a more active global policy framework.

Through increased recognition of refugee mental health, all of these global policy instruments could influence donors, program implementers, and health ministries. They could also spur constructive changes in the way that refugee and asylum applications are processed. For example, the UNHCR requires health screenings, but the mental health component could be strengthened so that any premigration or pre-resettlement need for psychiatric care could be factored into the choice of transit or destination country. Proposals consistent with this include the idea of a “health passport” for refugees (258). There are some precedents for this type of approach, such as the electronic health records system used by UNRWA for Palestinian refugees in several countries (259). This would require strong protections of privacy and confidentiality, given the stigma and discrimination associated with mental disorder and the risks faced by refugees regarding the potential misuse of health data (2, 260). But if handled correctly, and considered alongside other factors such as the existence of immigrant communities from the same country of origin, this information could ensure that refugees are placed in countries or regions where appropriate mental health care is available, along with social support. In this way it would help with the initiation of treatment or, in other cases, provide for continuity of care (one of the priority areas of the WHO's *Global Action Plan*). Such a system configuration could be adaptive to the specific needs of refugee populations, modulated to mental healthcare access or the health policy of a particular destination country. Critical to operationalizing this process is generating the needed partnerships and multimodal forms of data to match refugee populations based on their mental health needs and the appropriate phase of migration.

Improved global governance would also help influence national-level policy and implementation, especially the prioritization of refugee mental health within national mental health strategy. There are instructive examples available, as in Lebanon, where the Ministry of Public Health's new mental health plan includes refugees as an important vulnerable population requiring expanded services (181). This is necessary in a country where refugees make up a substantial portion of the population, but other countries have also moved in a similar direction. For example, soon after Syrian refugees were first welcomed to the country, Canada's Mental Health Commission issued a report specific to refugees, incorporating their potential needs into preexisting national mental health strategy (261). Problems with policy implementation for refugees nevertheless linger in most countries, but such prioritization at the national level is an important step forward in the push for increased financial commitments and service expansion in key destination countries.

The United States represents an important opportunity in this regard. Refugee resettlement dropped to historic lows in recent years, from a ceiling cap of 85,000 refugees during fiscal year 2016 to only 15,000 at the start of 2021 (262, 263). As a result, the United States is no longer the world's top country for refugee resettlement after having led the world for decades (264). A series of Executive Orders on immigration from 2017 to 2020 further slowed resettlement, which particularly affected the number of Syrian refugees coming to the country. From the years 2011 to 2019, a total of 21,725 Syrians arrived, with a high of 15,479 in 2016 and a low of 41 in 2018 (145, 265). The Biden administration made initial pledges to raise the annual refugee admission ceiling to 125,000 (266), confirmed in an Executive Order (267), although political realities have since complicated the situation (268). Rebuilding the US Refugee Admission Program may take years (266), but there are other steps the administration should consider in responding to the refugee crisis (269), including renewed and strengthened support of the WHO (270), which would signal the possibility of enhanced international leadership in this area.

Despite all of the challenges described in the preceding, particularly the problems associated with service scarcity and barriers to access, there are still important opportunities available for expanding and strengthening mental health services for refugees. The Syrian conflict should be a catalyst for these governance reforms to improve refugee mental health, as health is a fundamental human right recognized by the UN and WHO constitution. As others have emphasized, in some ways the current pandemic has provided an opportunity to revisit and revise public health systems on both a national (190) and international scale (270), including the pressing need to end longstanding neglect of the growing mental health burden (271). Now is the time for expansion and innovation in global health, guided by a strong vision of what is necessary to meet the needs of the most vulnerable. Migrant and refugee health is an important, indeed essential, part of this, and any and all developments in research, policy, and practice must grant refugee mental health a central place.

## Conclusion

While the conflict in Syria continues to appear intractable, the resulting public health emergency needs to be addressed in innovative ways. The expansion and improvement of mental health services is immediately necessary. What data there are describe an alarming crisis, with high prevalence and morbidity associated with mental disorder among Syrian refugees, met by relatively scarce and inaccessible services. These conditions hold in all of the primary destination countries in the region, including Turkey, Lebanon, Jordan, as well as in the countries outside the region that host refugees and resettlements, such as Germany. Most of these countries have applicable national legislation and policy frameworks, with some exceptions, but they all share problems with implementation. These national-level policies and service-level implementation efforts require strengthening, and the prioritization of refugee mental health within global policy instruments can help provide necessary guidance and influence.

## Author Contributions

KC, MAB, and TKM each made substantial contributions to the conceptualization, drafting, and revision of the manuscript. All the authors read and approved the final manuscript.

## Conflict of Interest

The authors declare that the research was conducted in the absence of any commercial or financial relationships that could be construed as a potential conflict of interest.

## Publisher's Note

All claims expressed in this article are solely those of the authors and do not necessarily represent those of their affiliated organizations, or those of the publisher, the editors and the reviewers. Any product that may be evaluated in this article, or claim that may be made by its manufacturer, is not guaranteed or endorsed by the publisher.

## Footnotes

^1^In the following, unless otherwise noted, the term “refugee” is used inclusively to refer to registered and non-registered refugees, asylum seekers, internally displaced persons, and displaced stateless persons.
